# Supplementary material for: A methodology for the design of experiments in computational intelligence with multiple regression models
Source: PeerJ. 2016 Dec 1;4:e2721. doi: 10.7717/peerj.2721 (PMC5136129; doi:10.7717/peerj.2721)
Supplement: Data S1 — Datailed results from UC Irvine Machine Learning Repository (Housing, Machine CPU, Wine Quality, Automobile and Parkinson) and the 3 Use Cases (Protein Corona, Gajewicz Metal Oxides and Aquatic Toxicity) [file peerj-04-2721-s001.zip › 6_UseCases/2_GajewiczMetalOxides/RF.details.csv.repeatedcv.split1.pdf]

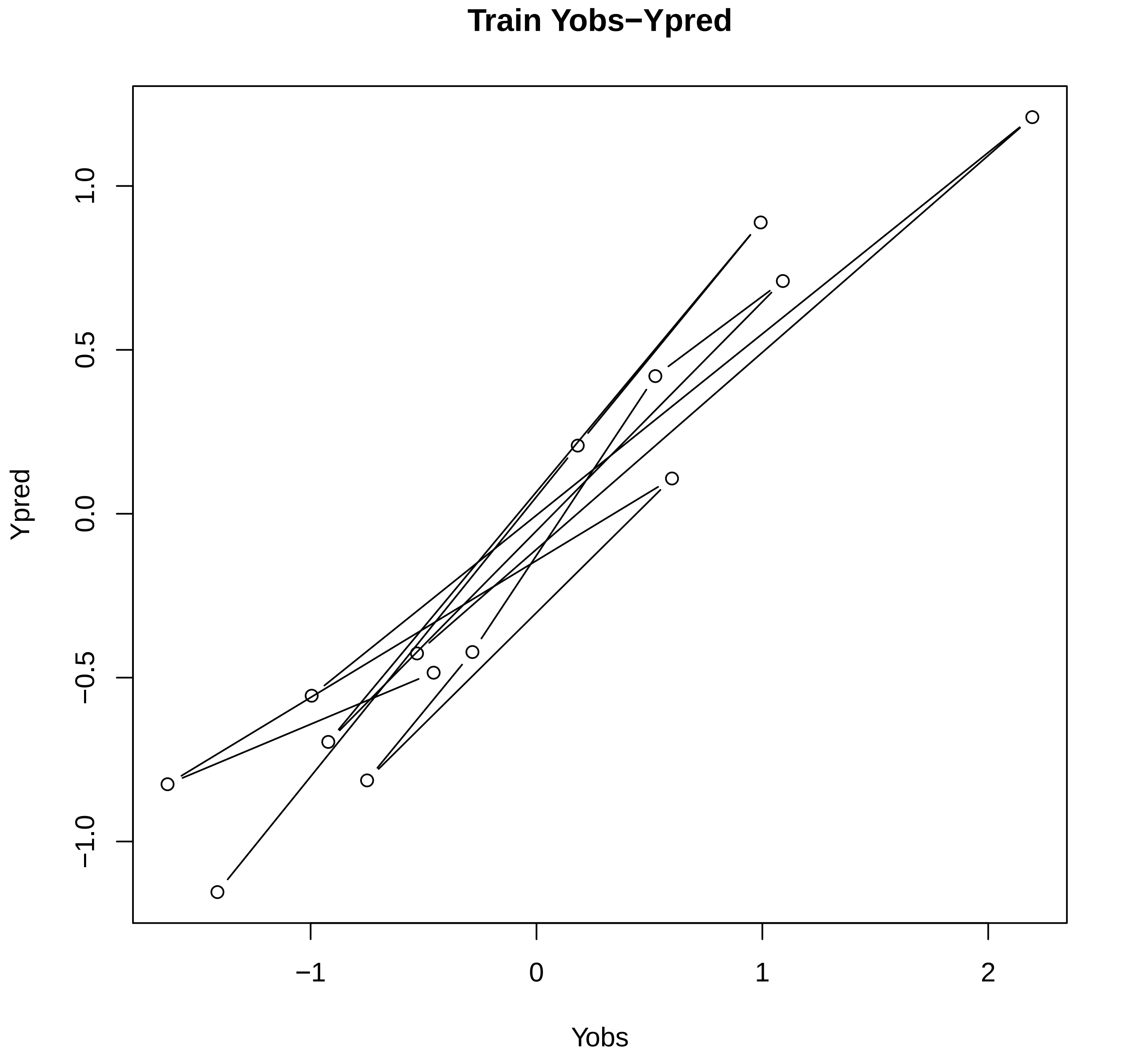
Train Yobs−Ypred
Ypred
Yobs
1.0
0.5
0.0
−0.5
−1.0
−1
0
1
2

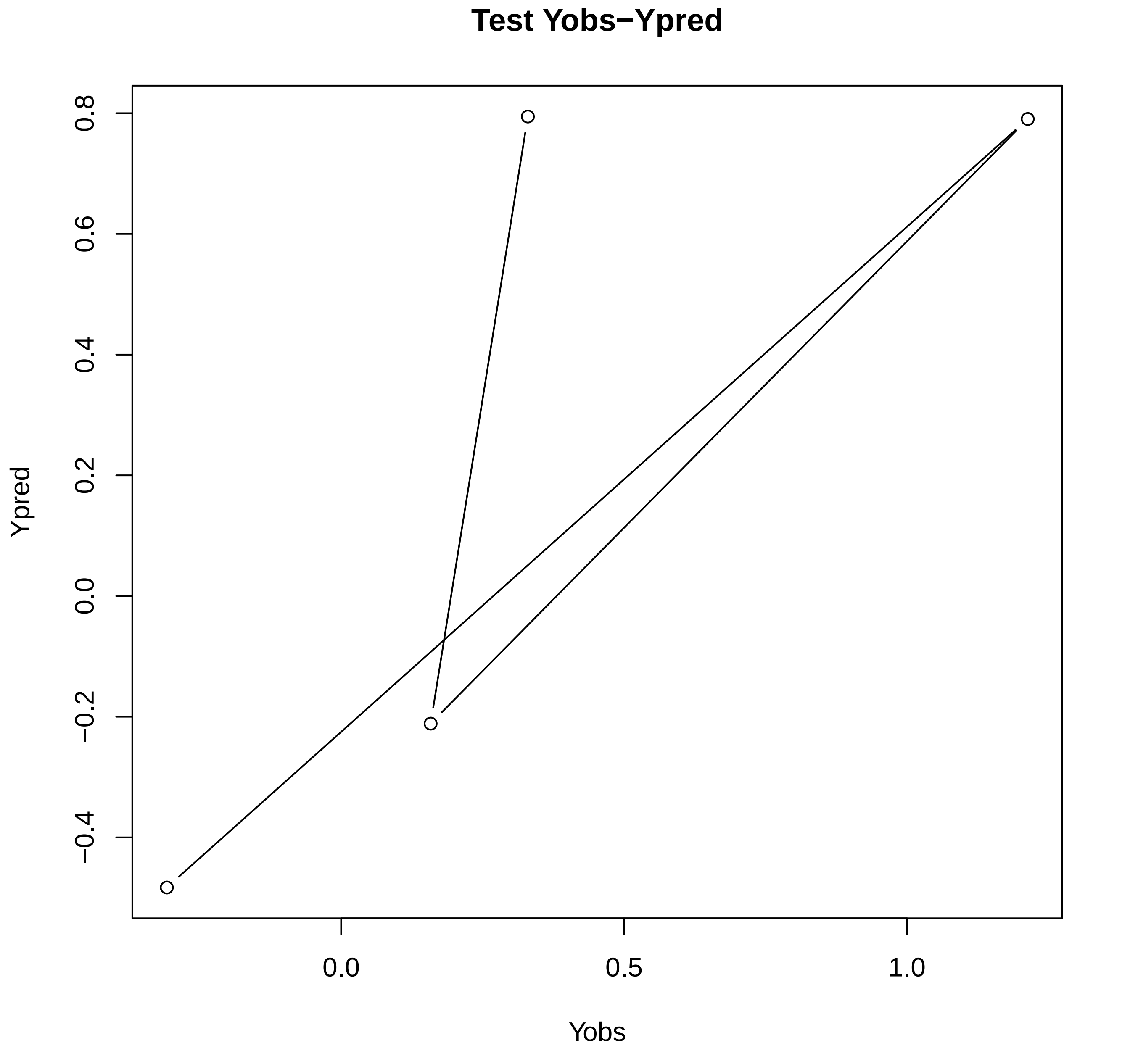
Test Yobs−Ypred
Ypred
Yobs
0.8
0.6
0.4
0.2
0.0
−0.2
−0.4
0.0
0.5
1.0

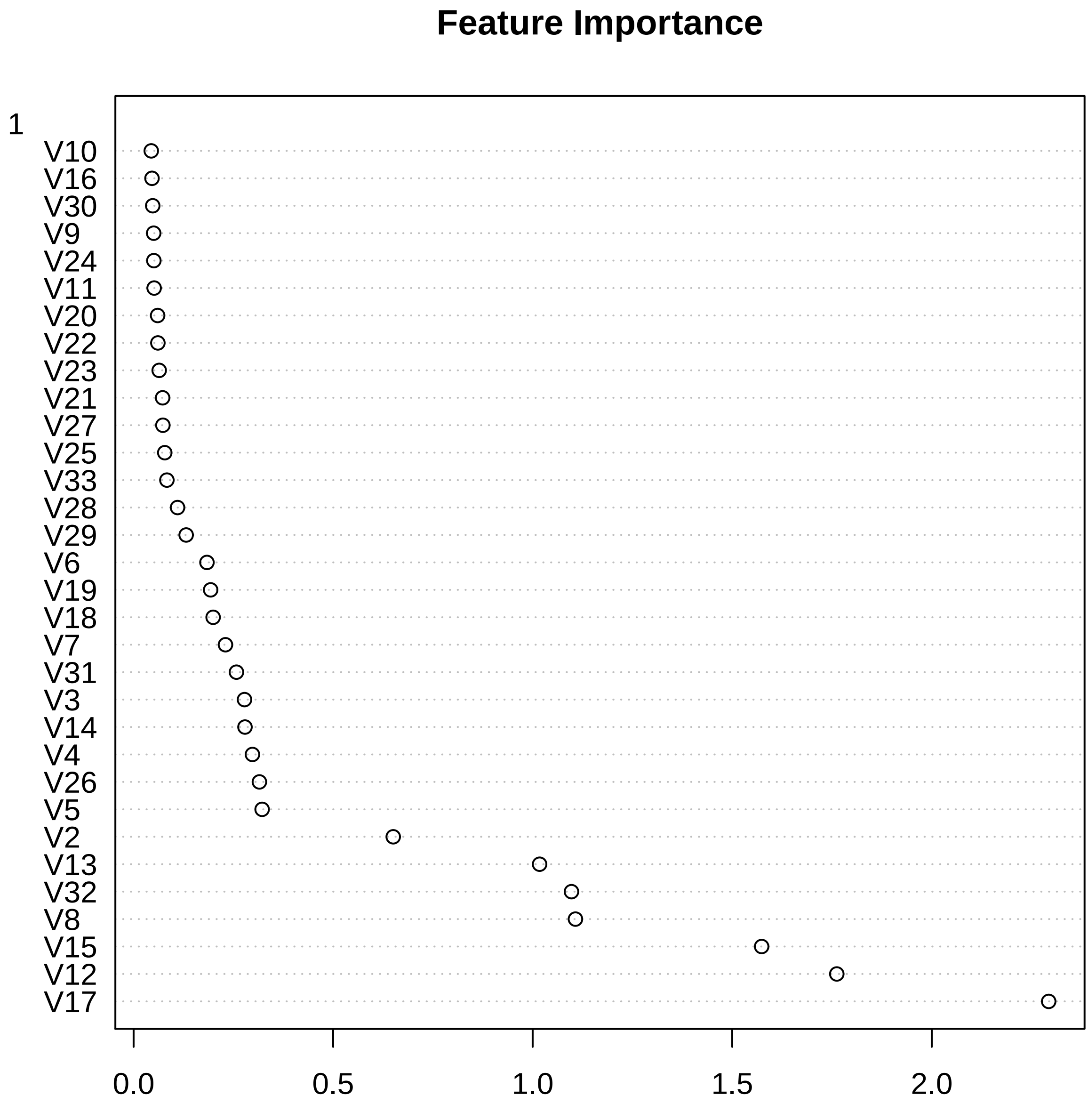

Feature Importance
1
V10
V16
V30
V9
V24
V11
V20
V22
V23
V21
V27
V25
V33
V28
V29
V6
V19
V18
V7
V31
V3
V14
V4
V26
V5
V2
V13
V32
V8
V15
V12
V17
0.0
0.5
1.0
1.5
2.0

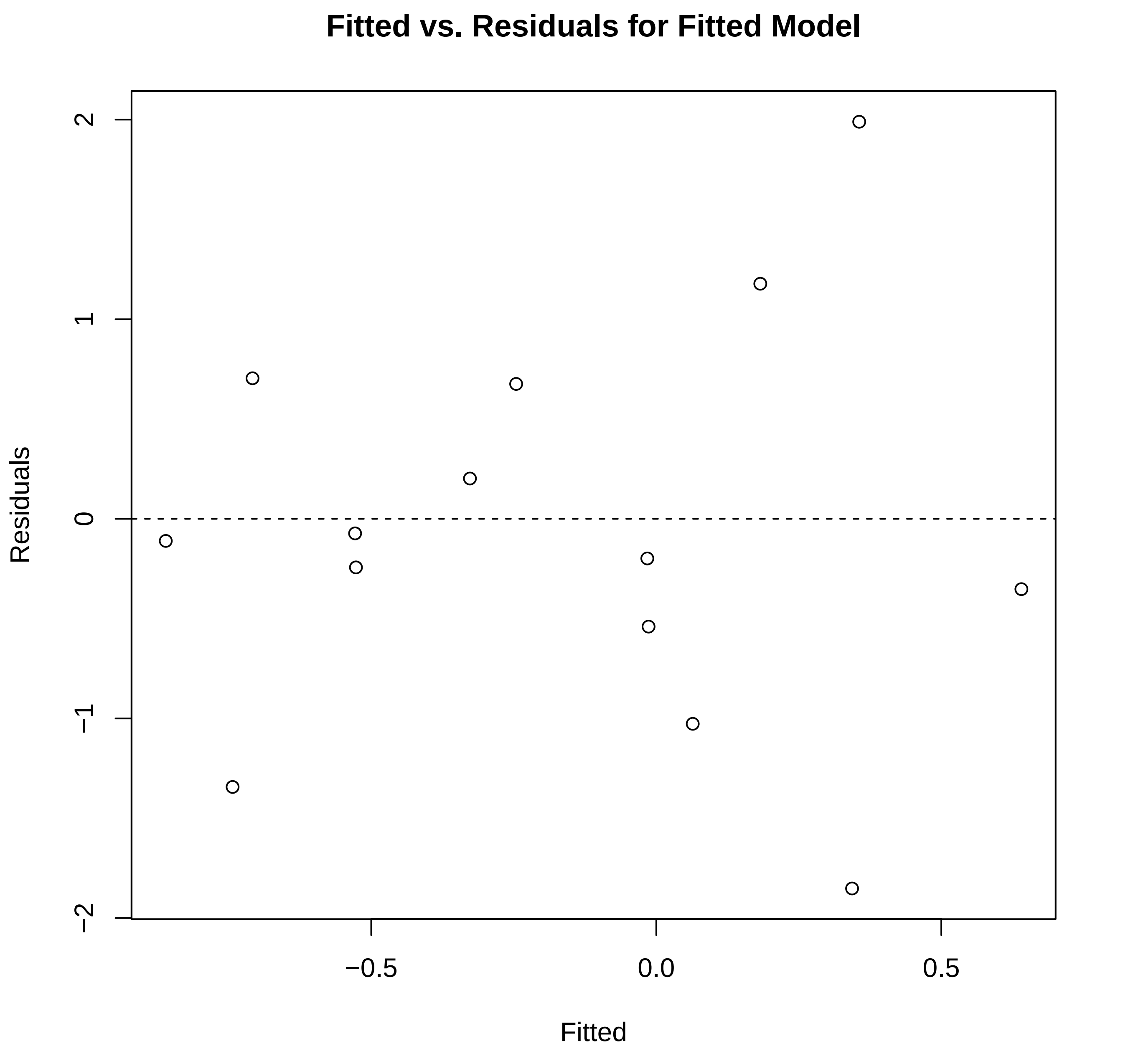
Fitted vs. Residuals for Fitted Model
2
1
0
−1
−2
Residuals
−0.5
0.0
0.5
Fitted
